# FOSL2 Positively Regulates TGF-β1 Signalling in Non-Small Cell Lung Cancer

**DOI:** 10.1371/journal.pone.0112150

**Published:** 2014-11-06

**Authors:** Junfeng Wang, Dawei Sun, Yanbo Wang, Fenghai Ren, Sainan Pang, Dandan Wang, Shidong Xu

**Affiliations:** The Department of Thoracic Surgery, Harbin Medical University Cancer Hospital, Harbin, China; Institute of Biomedical Sciences, Taiwan

## Abstract

Fos-related antigen 2 (FRA-2/FOSL2) belongs to the AP-1 transcription factor family. Although FOSL2 has been shown to be involved in diverse physiological and pathological processes, very little is known about the signalling pathways that regulate FOSL2 expression and the mechanisms of FOSL2 function. Here, we show that FOSL2 expression is regulated by TGF-β1 and that FOSL2 is required for TGF-β1-induced migration. We demonstrate that FOSL2 interacts with Smad3 *in vitro* and *in vivo* and thus up-regulates TGF-β1-induced signalling responses. Mechanistically, FOSL2 promotes P300 binding to Smad3 and the acetylation of Smad3 by P300. Furthermore, we show that the expression of FOSL2 correlates with activated Smad3 expression in clinical non-small cell lung cancer (NSCLC) samples. In summary, the present study indicates that FOSL2 facilitates TGF-β1-induced migration by interaction with Smad3 in NSCLC and suggests FOSL2 as a potential therapeutic target for NSCLC.

## Introduction

The TGF-β pathway controls diverse biological processes, including cell proliferation, differentiation, apoptosis, and migration [1]. Following the activation of heteromeric type II and type I serine-threonine kinase receptor complexes upon TGF-β ligand binding, intracellular signalling is initiated by phosphorylation of receptor activated Smad proteins (R-Smads) [2]. As transcriptional factors of the TGF-β pathway, phosphorylated Smad2/3 forms a complex with Smad4 and then translocates to the nucleus to regulate the transcription of TGF-β pathway target genes [3]. Cellular responses to TGF-β signalling are further influenced by the interaction of Smad proteins with cofactors (coactivators or corepressors) to modulate transcriptional activity [4].

Fos-related antigen 2 (FRA-2/FOSL2) belongs to the AP-1 transcription factor family, which includes the various isoforms of Fos and Jun [5]. The various FOS proteins play key roles in distinct developmental, physiological, and pathological processes [6], but these individual roles and the mechanisms involved are not yet clear. FOSL2 exerts a specific function in bone development [7] and appears to have selective physiological and pathological roles in diverse processes, including photoperiodic regulation [8], cancer [9], and fibrosis [10]. Several previous studies have indicated that FOSL2 plays a key role in the regulation of TGF-β pathway. For example, FOSL2 is overexpressed in systemic sclerosis (SSc) and acts as a novel downstream mediator of the profibrotic cytokine TGF-β [11]. In cardiac fibroblasts, FOSL2 as a transcriptional regulator may induce TGF-β expression [10]. Moreover, FOSL2 is necessary for TGF-β-induced lysyl oxidase-like 4 (LOXL4) expressions in the regulation of extracellular matrix (ECM) synthesis and remodeling [12]. However, it is unknown whether FOSL2 regulates TGF-β pathway in carcinogenesis.

The present study was initiated to examine the role of FOSL2 in TGF-β-induced migration. The expression of FOSL2 was increased following TGF-β treatment and was required for TGF-β1-induced migration. FOSL2 was also shown to bind to Smad3 to modulate TGF-β-induced signalling responses.

## Materials and Methods

### Ethics Statement

Patient information and samples were obtained with written informed consent. Each patient in this study gave written informed consent to publish these case details. The research was approved by the ethics committee of Harbin Medical University Cancer Hospital.

### Cell Lines, Cell Culture, and Transfection

The human lung adenocarcinoma cell line A549 and human embryonic kidney cell line 293T were purchased from American Type Culture Collection (ATCC) and maintained in DMEM supplemented with 10% foetal bovine serum (FBS) (Invitrogen) containing 100 units/ml penicillin and 100 units/ml streptomycin (Sigma) at 37°C with 5% CO_2_. For induction of EMT, A549 cells were cultured in 10% FBS for 24 hours and then maintained for 72 hours in serum-free medium in the presence of 2 ng/ml of TGF-β1 (R&D Systems). A549 cells were transfected using X-tremeGENE (Roche Applied Science), and HEK293T cells were transfected using Lipofectamine 2000 (Invitrogen) according to the manufacturer's directions. A small interfering RNA (siRNA) targeting human FOSL2 was transfected into cells for 24 hours using Lipofectamine RNAiMAX Reagent (Invitrogen).

### Plasmids and Antibodies

Myc-tagged Smad3, FLAG-tagged FOSL2, and HA-tagged p300 were constructed by standard subcloning. Full-length Smad3 was subcloned in-frame to the pGEX4T-1 vector to obtain GST fusion proteins. The 3TP-lux reporter plasmid was obtained from Dr Joan Massague of Sloan- Kettering Institute, New York, NY. Antibodies were purchased as follows: anti-FOSL2, anti-FLAG, anti-Myc, anti-HA, and anti-GAPDH from Sigma; anti-p300, anti-Smad3, and anti-GST from Santa Cruz; anti-acetylated-lysine from Cell Signaling Technology; anti-p-smad3 from Abcam; Alexa Fluor 488 donkey anti-mouse IgG and Alexa Fluor 546 donkey anti-rabbit IgG from Invitrogen. siRNAs for FOSL2 and the negative control were obtained from Dharmacon.

### Immunoprecipitation and Western Blotting Analysis

At 24 hours after transfection, cell lysates were harvested using lysis buffer (50 mM Tris, pH 8.0, 150 mM NaCl, 5 mM EDTA, 50 mM NaF, and 0.1% NP-40) and incubated with protein A or G Sepharose beads and the appropriate antibodies for 2 h at 4°C. After extensive washes, the immunoprecipitated proteins were boiled in protein sample buffer for 5 min and then separated by SDS-PAGE, transferred onto PVDF membranes (Millipore), and detected by western blotting analysis.

### GST Pull-down Assay

GST and GST-Smad3 fusion proteins were expressed in BL21 cells and purified according to the manufacturer's instructions (GE Healthcare Life Science). FLAG-tagged FOSL2 constructs were expressed in HEK 293T cells. Whole-cell protein lysates were harvested after 48 hours using cell lysis buffer, precleared using glutathione Sepharose beads, and then incubated with either GST-Smad3 or control GST for 2 hours at 4°C. The beads were washed five times with GST-binding buffer, eluted in 40 µL of 2×SDS sample buffer, and then detected by immunoblotting.

### Transcription Reporter Assay

Cells were treated with or without 2 ng/ml TGF-β1 at 20 hours after transfection. The cells were then harvested and analysed with the Dual Luciferase Reporter Assay system (Promega). All assays were performed in triplicate, and all values were normalised for transfection efficiency against *Renilla* luciferase activities.

### Real-time RT-PCR (qRT-PCR)

Total RNAs were obtained using the TRIzol reagent (Invitrogen). Reverse transcription of RNA was performed using the ImProm-II reverse transcription system (Promega) according to the manufacturer's instructions. Quantitative reverse transcriptase (qRT)-PCR was performed using an ABI PRISM 7500 Sequence Detector System (Applied Biosystems) with gene-specific primers for p21 and PAI-1.

### Immunofluorescence

A549 cells were cultured in a 6-well plate and treated with TGF-β1 (2 ng/ml). The cells were then fixed with 4% paraformaldehyde, permeabilised with 0.1% Triton X-100, and incubated with primary antibodies against FOSL2 or Smad3 for 1 h at 37°C. The cells were then incubated with Alexa Fluor 488 or Alexa Fluor 546 antibodies for 30 min at 37°C. The fluorescent images were captured using a confocal laser-scanning microscope.

### Immunohistochemistry

Tissue specimens were embedded in paraffin. The sections were deparaffinised in xylene and rehydrated in an ethanol gradient. After antigen retrieval, the sections were treated with 3% H_2_O_2_ for 10 min, followed by 5% bovine serum albumin (BSA) for 30 min. The sections were then incubated with primary antibodies. The visualisation of antibody binding was performed using DAB staining. The nuclei were stained with haematoxylin. The immunostaining results were independently assessed by two pathologists.

### Patients

Lung cancer specimens (n = 57) were collected from patients with NSCLC in Harbin Medical University Cancer Hospital from 2009 to 2012. The tissues were stored at −80°C until use. All samples were from patients who had not undergone preoperative radiotherapy or chemotherapy. The pathological staging of the 57 tumours was performed according to the tumour-node-metastasis (TNM) staging system.

### Cell Migration Assay

Migration assays were performed with 8-µm filters (BD Biosciences). Each well was loaded with ∼1×10^5^ cells. After incubation for 16 h, the cells passing through the filter into the bottom wells were fixed in formalin and stained with Crystal Violet. The cells in 10 randomly selected fields (200×) from each well were counted.

### Statistical Analyses

All statistical analyses were performed using the SPSS17.0 software. Other statistical analyses were performed using a Student t test. The Kaplan-Meier survival analysis for evaluating overall survival time was performed by the log-rank test. The data are shown as the mean ± SD from 3 independent assays. A statistically significant difference was considered when *P*<0.05.

## Results

### FOSL2 is required for TGF-β1-induced migration

**Figure 1:**
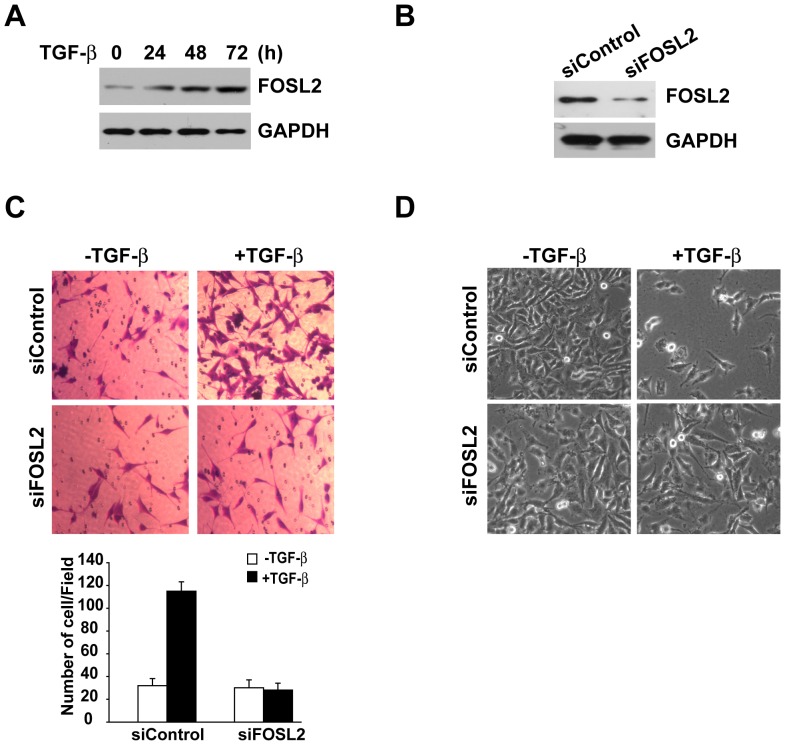
FOSL2 is required for TGF-β1-induced migration. (A) A549 cells were incubated with TGF-β1 (2 ng/ml) for the indicated times, and the cells were collected for western blot analysis. GAPDH was used as a loading control. (B) FOSL2 levels were examined by western blotting in FOSL2-siRNA and sicontrol A549 cells. GAPDH was also determined as a loading control. (C) Cell migration was measured using Transwell assays in sicontrol and siFOSL2 A549 cells with or without TGF-β1 (2 ng/ml) treatment. The cells migrating to the lower surface of the Transwell filters were photographed (top) and counted (bottom). (D) siControl and siFOSL2 A549 cells were incubated with or without 2 ng/ml of TGF-β1 for 72 h. Phase-contrast microscopy shows the cell morphological changes.

To begin to explore the possibility that FOSL2 expression is regulated by TGF-β1, we treated A549 cells with TGF-β1 over a time course spanning 24 h to 72 h and performed a western blot analysis. The results showed that FOSL2 levels increased substantially in response to TGF-β1 treatment in this cell line (Figure 1A). The maximal FOSL2 expression levels at 72 h were approximately 4 times those of the control basal level (Figure 1A). Next, we tested whether FOSL2 participates in TGF-β1-induced migration. To explore this possibility, we knocked down FOSL2 expression in A549 cells, and a western blot analysis indicated that FOSL2 levels were significantly decreased compared to the control cells (Figure 1B). Then, we examined the regulatory effect of FOSL2 on the migratory ability of A549 cells using a Transwell migration assay. TGF-β1 treatment dramatically promoted the migration of A549 cells, whereas knocking down FOSL2 abolished cell migration in the presence of TGF-β1 (Figure 1C). In addition, we also examined the morphological changes of A549 cells after exposure to TGF-β1. In the absence of TGF-β1, the cells maintained a classic cobblestone epithelial morphology and growth pattern, but the cells adopted a more fibroblast-like morphology and reduced their cell-cell contact after TGF-β1 stimulation; however, this effect was inhibited by FOSL2 depletion (Figure 1D).

### FOSL2 interacts with Smad3

**Figure 2:**
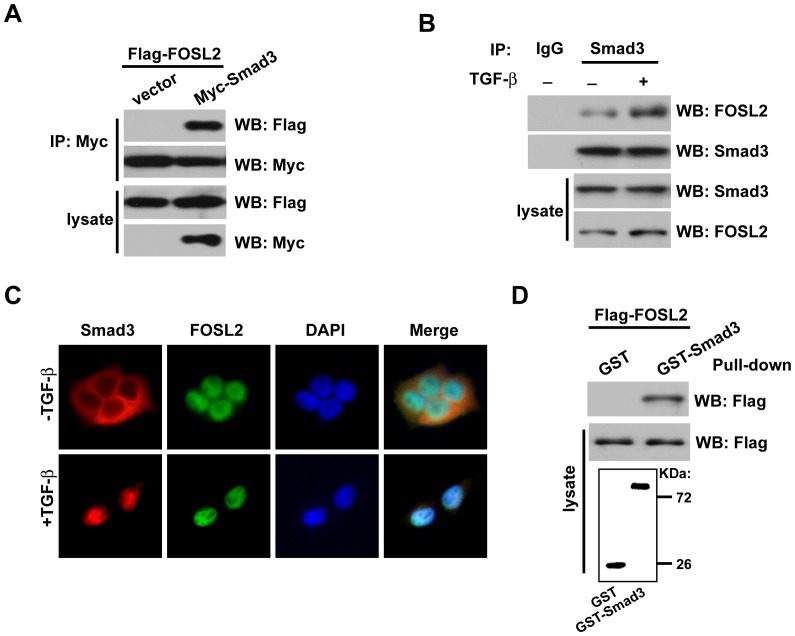
FOSL2 interacts with Smad3. (A) FOSL2 interacts with Smad3 in vivo. FLAG-FOSL2 and Myc-Smad3 were co-transfected into HEK293T cells. Cell lysates were harvested, and IP was performed with an anti-Myc antibody. FOSL2 and Smad3 were detected from the immunoprecipitates by western blotting with the indicated antibodies. (B) The association between endogenous FOSL2 and Smad3 in A549 cells with TGF-β1 (2 ng/ml) treatment. Immunoprecipitation was performed using an anti-IgG antibody or anti-Smad3 antibody. (C) Subcellular colocalisation of FOSL2 and Smad3 in A549 cells in the presence TGF-β1 (2 ng/ml). The nuclei were stained with DAPI. (D) Equivalent amounts of GST-Smad3 or GST alone were incubated with cell lysates from HEK 293T cells overexpressing Flag-FOSL2; 10% of the input was run on the gel as a control. The GST-Smad3 was detected by staining gels with Coomassie Blue.

Because Smad2/3 proteins are the transcriptional effectors of TGF-β1 signalling, we examined whether the inhibition of TGF-β1-induced migration by FOSL2 depletion was mediated by interaction with these Smads. To test whether there is an interaction between FOSL2 and Smad3 in vivo, we used cell lysates from 293T cells transfected with expression plasmids for Flag-tagged FOSL2 and Myc-tagged Smad3 and found that FOSL2 could be co-precipitated with Smad3 (Figure 2A), whereas an interaction between FOSL2 and Smad2 was not observed (data not shown). We then determined whether endogenous FOSL2 and Smad3 also interact. As shown in Figure 2B, endogenous FOSL2 associated with Smad3 in A549 cells, and the interaction was notably apparent in the presence of TGF-β1. Moreover, FOSL2 colocalised with Smad3 in the presence of TGF-β1 (Figure 2C). To determine whether FOSL2 directly interacts with Smad3, we performed GST pull-down assays. Equivalent amounts of GST-Smad3 or GST protein alone were incubated with Flag-tagged FOSL2. FOSL2 directly interacted with Smad3, but no interaction of Smad3 was apparent with the GST protein (Figure 2D).

### FOSL2 modulates TGF-β1-induced signalling responses

**Figure 3:**
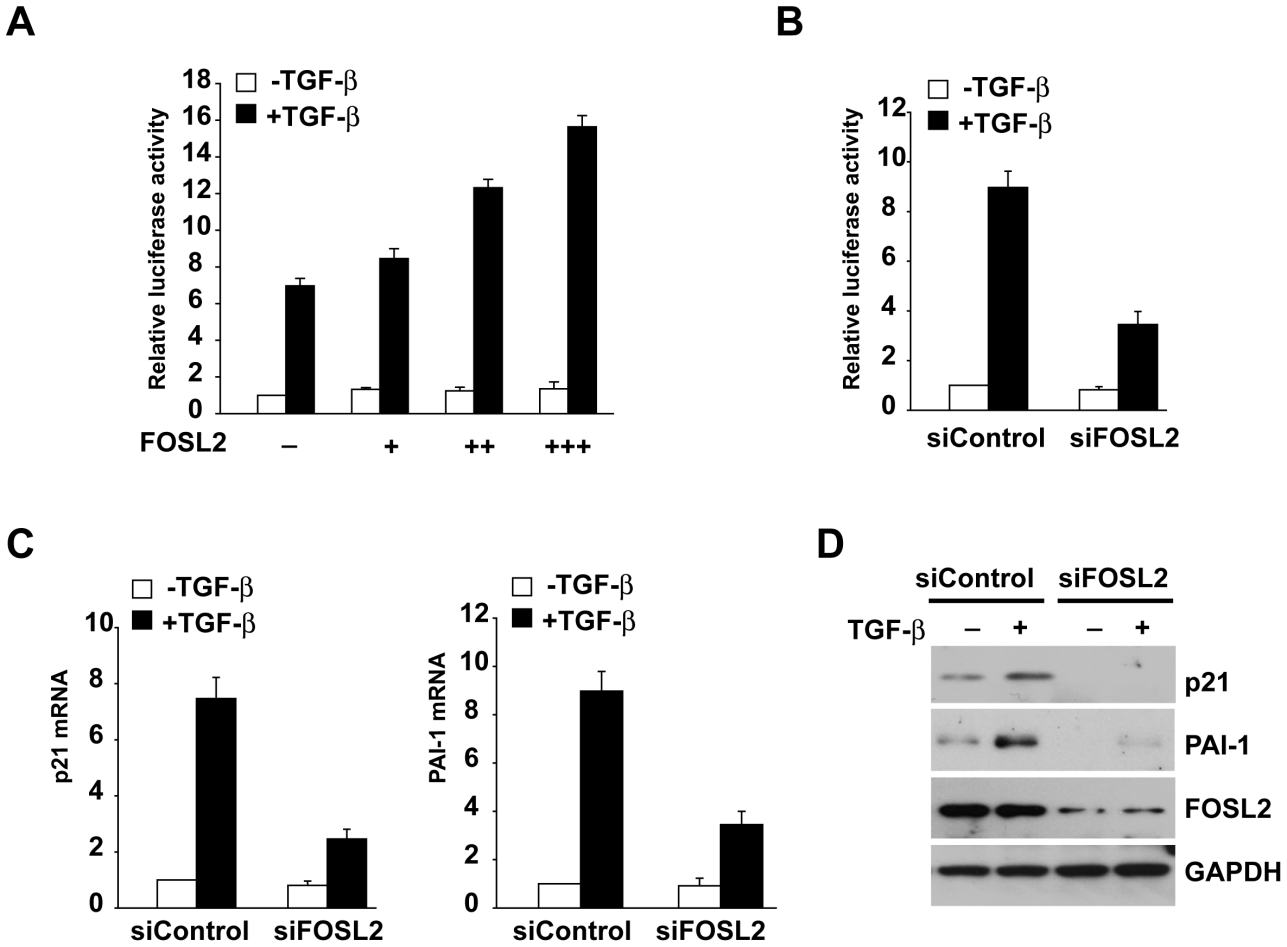
FOSL2 modulates TGF-β1-induced signalling responses. (A) HEK 293T cells were transfected with the reporter p3TP-Lux plasmid harbouring Smad3 in the absence and presence of FOSL2, as indicated. The cells were harvested at 36 h after transfection and assayed for luciferase activity. The data are the means ± s.d. (B) A549 cells were transfected with siFOSL2 and control scrambled siRNA. After 24 h, the cells were transfected with p3TP-Lux and Smad3, as indicted. Luciferase activity was assayed after a further 24 h. The data are means ± s.d. (C) A549 cells transfected with siFOSL2 and control scrambled siRNA were stimulated with 2 ng/ml of TGF-β1 for 4 h. Total mRNAs were analysed by qRT-PCR using primers specific for p21 and PAI-1. The data are means ± s.d. (D) A549 cells transfected with siFOSL2 and control scrambled siRNA were stimulated with 2 ng/ml of TGF-β1 for 4 h and then harvested to produce cell lysates. The expression levels of the indicated proteins were examined by western blotting with the appropriate antibodies, as indicated.

We next investigated the effect of FOSL2 expression on TGF-β1-dependent transcriptional responses using the 3TP-Lux reporter. Co-expression of FOSL2 gradually resulted in an up-regulation of Smad3-induced reporter activity (Figure 3A). Conversely, the reporter activity was decreased in A549 cells transfected with FOSL2 siRNA (siFOSL2) relative to the control cells transfected with scrambled siRNA(Figure 3B), confirming that endogenous FOSL2 regulates Smad3 activity.

We then evaluated the effect of FOSL2 knockdown on the endogenous expression of TGF-β1/Smad3 target genes. In A549 cells, TGF-β1 treatment induced the rapid expression of p21 and PAI-1 mRNAs, and knockdown of FOSL2 significantly attenuated these effects (Figure 3C). Consistently, TGF-β1-induced p21 and PAI-1 expression at the protein level was inhibited in A549 cells following FOSL2 depletion (Figure 3D).

### FOSL2 promotes P300 binding to Smad3 and the acetylation of Smad3 by P300

**Figure 4:**
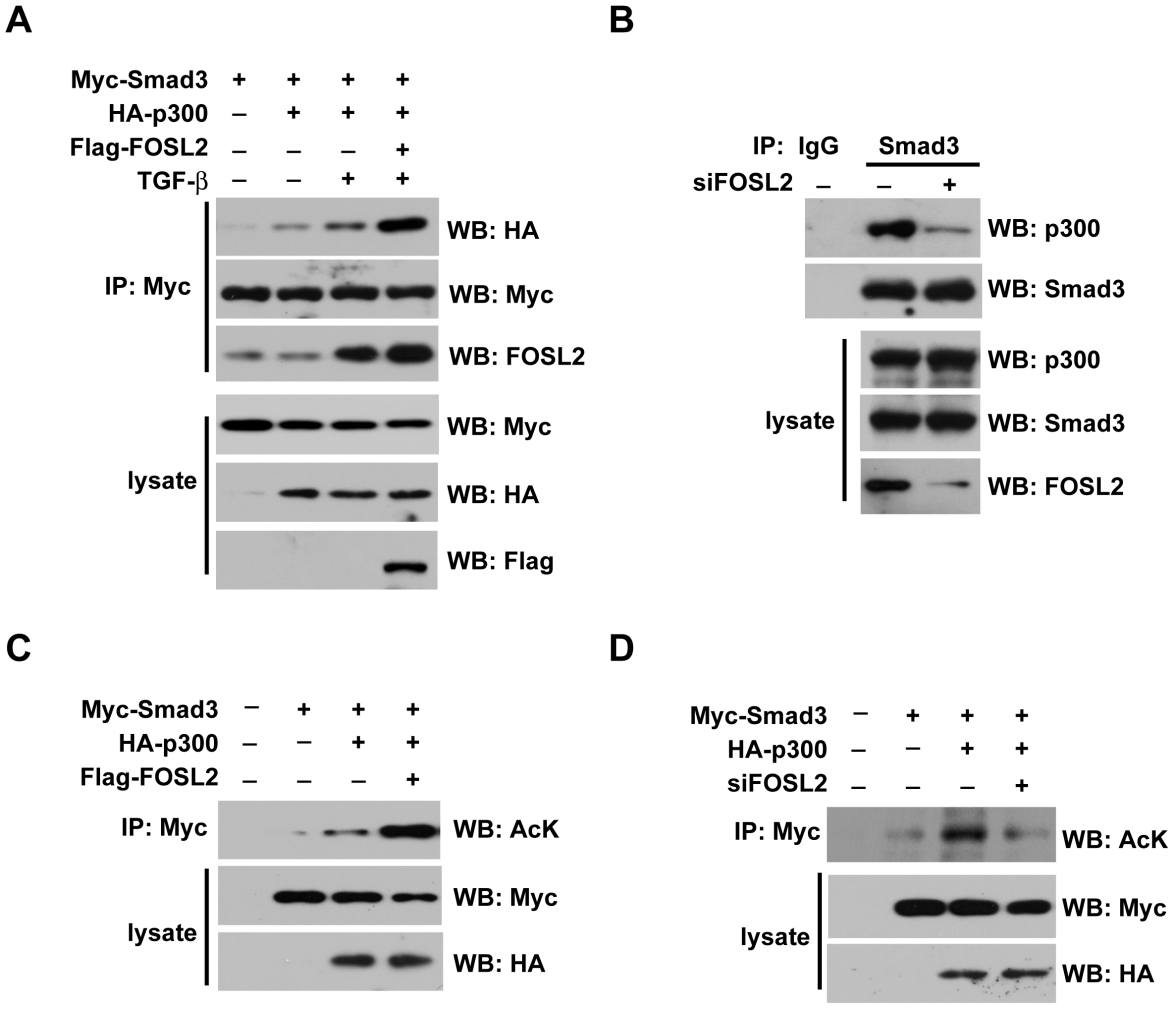
FOSL2 promotes P300 binding to Smad3 and the acetylation of Smad3 by P300. (A) HEK293T cells were cotransfected with expression plasmids for HA-p300 and FLAG-FOSL2, together with Myc-Smad3, as indicated, with or without with TGF-β1 (2 ng/ml) treatment. Smad3-bound p300 was immunoprecipitated with an anti-Myc antibody and detected by western blotting with an anti-HA antibody. (B) A549 cells were transfected with siFOSL2. After 24 h, the cells were treated, as indicated, with TGF-β1 (2 ng/ml) treatment. (C) HEK293T cells were transiently transfected with the indicated plasmids. The cells were incubated in the presence of TSA (5 µM) for 20 h. Cell lysates were immunoprecipitated (IP) with an anti-Myc antibody, and acetylated Smad3 (Ac-Smad3) was detected by western blotting with an anti-acetylated lysine antibody. (D) A549 cells were transiently transfected with the indicated plasmids. The experiments were performed as described in Fig. 4C.

It is well known that CBP/p300 cooperates with the Smad complex to regulate TGF-β target gene transcription, which stabilises the transcriptional activity of the Smad complex and thus increases the duration of TGF-β signalling. To further explore the mechanism of the regulation of FOSL2 in TGF-β signalling, we examined whether FOSL2 affects the TGF-β1-induced formation of the Smad3/p300 complex. The interaction between Smad3 and p300 was induced by TGF-β1 stimulation, and this interaction was profoundly increased by the ectopic expression of FOSL2 (Figure 4A). In contrast, the Smad3 and p300 interaction was attenuated by the knockdown of FOSL2 (Figure 4B). Because Smad3 acetylation by p300 positively regulates its transcriptional activity, we thus examined whether FOSL2 is involved in this process. To test this possibility, 293T cells were transfected with Myc-Smad3 alone or together with p300 or FOSL2. As shown in Figure 4C, p300 induced the acetylation of Smad3, and FOSL2 overexpression significantly improved this acetylation. Furthermore, endogenous FOSL2 depletion suppressed the acetylation of Smad3 by p300 in A549 cells (Figure 4D).

### Correlation of FOSL2 and activated Smad3 expression in NSCLC tumours

**Figure 5:**
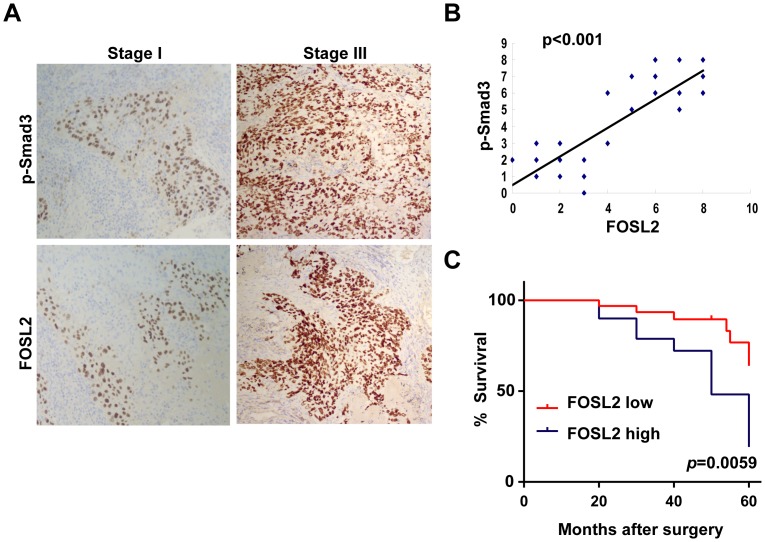
Correlation of FOSL2 and activated Smad3 expression in NSCLC tumours. (A) Immunohistochemical analysis of FOSL2 and p-Smad3 in 57 NSCLC samples. (B) FOSL2 expression is positively correlated with p-Smad3 expression in NSCLC samples. Semiquantitative scoring was performed (Pearson correlation test; r = 0.858, p<0.001). Note that some of the dots on the graphs represent more than one specimen (some scores overlapped). (C) Kaplan-Meier survival curves for NSCLC patients with FOSL2 higher or lower expression. The difference in postoperative survival between NSCLC patients with FOSL2 higher expression and with FOSL2 lower expression was greatly significant (*P* = 0.0059 by log-rank test).

To further investigate the clinical relationship between FOSL2 and TGF-β1 signalling, the expression of FOSL2 and p-Smad3 in 57 NSCLC samples were examined by immunohistochemical methods, and correlations of the proteins were evaluated. Out of 57 cases, 35 were pathological Stage I, and 22 were in pStage III. Of the 35 cases in pStage I, 31 (88.6%) showed a lower expression of the FOSL2 and p-Smad3 proteins; however, 18 of the 22 cases (81.8%) in pStage III showed a higher expressions of both proteins (Figure 5A). Moreover, FOSL2 expression was positively correlated with p-Smad3 staining (P<0.001) (Figure 5B). The Kaplan-Meier survival curves showed that patients with FOSL2 higher expression were at a notably greater risk of an earlier death than those with FOSL2 lower expression (P = 0.0059) (Figure 5C). Therefore, these results indicated that FOSL2 expression in lung cancer tissue correlates with postoperative relapse and survival of lung cancer patients.

## Discussion

Our results demonstrate that FOSL2 up-regulation is necessary for TGF-β1-induced migration. The inhibition of FOSL2 expression prevented TGF-β1-induced migration and the associated morphological changes. Collectively, these results suggest that FOSL2 is an important component of a regulatory network in TGF-β1-induced migration.

Smad3 is a key signal transducer in TGF-β1-induced signalling pathways [13]. In an effort to further elucidate the relationship between FOSL2 and Smad3, we identified FOSL2 as a co-factor for Smad3. How FOSL2 functions in cells has not been characterised to date [14]. We first validated the physical interactions of FOSL2 with Smad3. In the nucleus, Smad oligomers recruit the transcriptional co-activators p300/CBP [15]–[17], which are structurally related proteins with histone acetyltransferase (HAT) activity. Acetylation is a post-translational modification of proteins, with histones being the best known example [18]. Smad3 is a direct target of the transcriptional co-activators p300/CBP, and this acetylation is stimulated by TGF-β [19]. However, it is not clear whether there are other proteins that facilitate this process. In this report, we demonstrate that FOSL2 promotes P300 binding to Smad3 and Smad3 acetylation by P300, suggesting that FOSL2 plays a positive role in regulating TGF-β signalling.

Metastasis is a highly complex process that involves the escape of tumour cells from the primary tumour mass, migration and invasion through basal membranes and connective tissues, entry into the vascular or lymphatic system, survival in the circulation, extravasation at different organ sites (including attachment to endothelial cells and diapaedesis across the endothelium), and proliferation to form a distant metastasis [20]–[22]. Previous results have shown that FOSL2 overexpression leads to an increased invasive potential in breast cancer cells, but the mechanism has not been elucidated thus far [23]. Given that TGF-β1 can utilise various programmes to promote cancer metastasis through its effects on the tumour microenvironment, enhanced invasive properties, and inhibition of immune cell function, our findings also reveal that FOSL2 may increase invasive potential through the TGF-β pathway. Moreover, our clinical results of a correlation between FOSL2 and activated Smad3 expression in NSCLC tumours further confirm this conclusion. These findings highlight the contribution of FOSL2 to non-small cell lung tumourigenesis and raise the possibility of inhibiting FOSL2 in NSCLC therapy.

In summary, we provide the first evidence that FOSL2 facilitates TGF-β1-induced migration in NSCLC cells by interaction with Smad3 and thus promotes P300 binding to Smad3 and Smad3 acetylation by P300, events that may contribute to the development of NSCLC and suggest FOSL2 as a potential therapeutic target in NSCLC.
